# 2-Methyl-pentanoyl-carnitine (2-MPC): a urine biomarker for patent *Ascaris lumbricoides* infection

**DOI:** 10.1038/s41598-020-72804-y

**Published:** 2020-09-25

**Authors:** Ole Lagatie, Ann Verheyen, Stijn Van Asten, Maurice R. Odiere, Yenny Djuardi, Bruno Levecke, Johnny Vlaminck, Zeleke Mekonnen, Daniel Dana, Ruben T’Kindt, Koen Sandra, Rianne van Outersterp, Jos Oomens, Ronghui Lin, Lieve Dillen, Rob Vreeken, Filip Cuyckens, Lieven J. Stuyver

**Affiliations:** 1 Janssen Global Public Health, Janssen R&D, Turnhoutseweg 30, 2340 Beerse, Belgium; 2 Discovery Sciences, Janssen R&D, Turnhoutseweg 30, 2340 Beerse, Belgium; 3 Centre for Global Health Research, Kenya Medical Research Institute, P. O. Box 1578, Kisumu, 40100 Kenya; 4 Department of Parasitology, Faculty of Medicine, Universitas Indonesia, Jakarta, Indonesia; 5 Department of Virology, Parasitology and Immunology, Ghent University, Salisburylaan 133, 9820 Merelbeke, Belgium; 6 School of Medical Laboratory Sciences, Jimma University, Jimma, Ethiopia; 7 Research Institute for Chromatography, President Kennedypark 26, 8500 Kortrijk, Belgium; 8 FELIX Laboratory, Faculty of Science, Radboud University, Toernooiveld 7, 6525 ED Nijmegen, The Netherlands; 9 Janssen R&D, Welsh & McKean Road, Spring House, PA 19477-0776 USA

**Keywords:** Diagnostic markers, Parasitic infection

## Abstract

Infections with intestinal worms, such as *Ascaris lumbricoides,* affect hundreds of millions of people in all tropical and subtropical regions of the world. Through large-scale deworming programs, World Health Organization aims to reduce moderate-to-heavy intensity infections below 1%. Current diagnosis and monitoring of these control programs are solely based on the detection of worm eggs in stool. Here we describe how metabolome analysis was used to identify the *A. lumbricoides*-specific urine biomarker 2-methyl pentanoyl carnitine (2-MPC). This biomarker was found to be 85.7% accurate in determining infection and 90.5% accurate in determining a moderate-to-heavy infection. Our results also demonstrate that there is a correlation between 2-MPC levels in urine and *A. lumbricoides* DNA detected in stool. Furthermore, the levels of 2-MPC in urine were shown to rapidly and strongly decrease upon administration of a standard treatment (single oral dose of 400 mg albendazole). In an *Ascaris suum* infection model in pigs, it was found that, although 2-MPC levels were much lower compared to humans, there was a significant association between urinary 2-MPC levels and both worm counts (*p* = 0.023) and the number of eggs per gram (epg) counts (*p* < 0.001). This report demonstrates that urinary 2-MPC can be considered an *A. lumbricoides*-specific biomarker that can be used to monitor infection intensity.

## Introduction

Soil-transmitted helminths (STHs), such as *Ascaris lumbricoides*, *Trichuris trichiura,* and the hookworm species *Ancylostoma duodenale, Ancylostoma ceylanicum* and *Necator americanus,* pose a major threat to public health in large parts of the world. Often also *Strongyloides stercoralis* is included in this list, but this nematode has some peculiar differences compared to the other STHs, such as the fact that it puts infected subjects at risk of a fatal syndrome^1^. The STH-attributable morbidity is mainly associated with moderate-to-heavy intensity (M&HI) infections, children and women of childbearing age being at highest risk^2^. Control of the infection pressure is mainly approached by means of so-called preventive chemotherapy (PC) programs in which anthelminthic drugs (e.g. mebendazole or albendazole) are administered to school-age children, regardless of their infection status^3^. The goal of the World Health Organization (WHO) is to reduce the prevalence of M&HI infections in preschool and school-aged children to below 1% by 2020^2^. Global control efforts for soil-transmitted helminthiasis are an essential part of the Sustainable Development Goals (SDGs) put forward by the WHO where it contributes to achieve goal 3: good health and well-being. To enable proper monitoring of the current programs and support decision-taking, there is a growing need for improved diagnostics^4^. Current procedures that are being used in the field are based on the detection and quantification of STH eggs in a stool smear using a compound microscope, the so-called Kato-Katz thick smear^5–7^. However, this microscopic examination lacks standardization, shows day-to-day and intra-stool variability and is not ideal for screening large number of samples^8^. This becomes particularly apparent when sample size increases to demonstrate a drop below 1% prevalence, as was already experienced for *Schistosoma mansoni*^9^. Kato-Katz is also time-consuming and requires trained personnel and specific laboratory infrastructure. The STH-community has begun exploring the introduction of new diagnostic tools for non-stool-based biomarkers, similar to what was done for schistosomiasis with the introduction of the urine-based point-of-care circulating cathodic antigen (POC-CCA) test^9^. Target Product Profiles (TPPs) describing the specific requirements for such new STH diagnostic approaches required for different program use cases were published. These TPPs indicate that the identification of non-stool-based biomarkers will be essential to enable the development of diagnostic tools for making decisions on whether or not to stop interventions (use case 3) or to verify sustained break in transmission (use case 4)^4^.

In a search for such non-stool-based biomarkers for STH infection, 2-methyl butyramide (2-MBA) and 2-methyl valeramide (2-MVA) were previously identified in urine as promising biomarkers for infection with *A. lumbricoides*^10^. However, these findings could not be confirmed in a more recent study on *A. lumbricoides-*infected subjects from Indonesia^11^.

In this study, we used an untargeted liquid chromatography (LC)-mass spectrometry (MS)-based metabolomics approach to identify potential biomarkers in plasma and urine of *A. lumbricoides* infected human subjects. The most promising newly discovered biomarker was evaluated in different sample sets from different endemic regions. Furthermore, pigs experimentally infected with *A. suum* were used to confirm that the presence of this biomarker is closely linked to *Ascaris* infection.

## Results

To identify novel biomarkers in body fluids of *A. lumbricoides* infected subjects, urine and plasma of both *A. lumbricoides* infected and non-infected subjects from Kenya, as well as from a non-endemic Belgian population were subjected to metabolomic profiling. An overview of all samples used, is detailed in Table 1.

**Table 1:** Overview of samples used in metabolomic profiling.

Group	Origin	n	Median fecal egg counts (epg; min–max)
*A. Lumbricoides* infected	Kenya	38	774 (67–24,000)
Endemic not *Al* infected	Kenya		
*T. trichiura* infected		37	60 (12–2,184)
*S. mansoni* infected		39	144 (12–1,320)
Not infected		42	n.a
Non-endemic controls	Belgium	40	n.d

All samples were divided in two groups: one infected with only *A. lumbricoides* and one not infected with *A. lumbricoides*. For the latter group, individuals infected with *T. trichiura*, hookworm and *S. mansoni* based on microscopic egg counting by the Kato-Katz thick smear are listed.

epg: eggs per gram of stool; n.a.: not applicable; n.d.: not determined.

**Figure 1:**
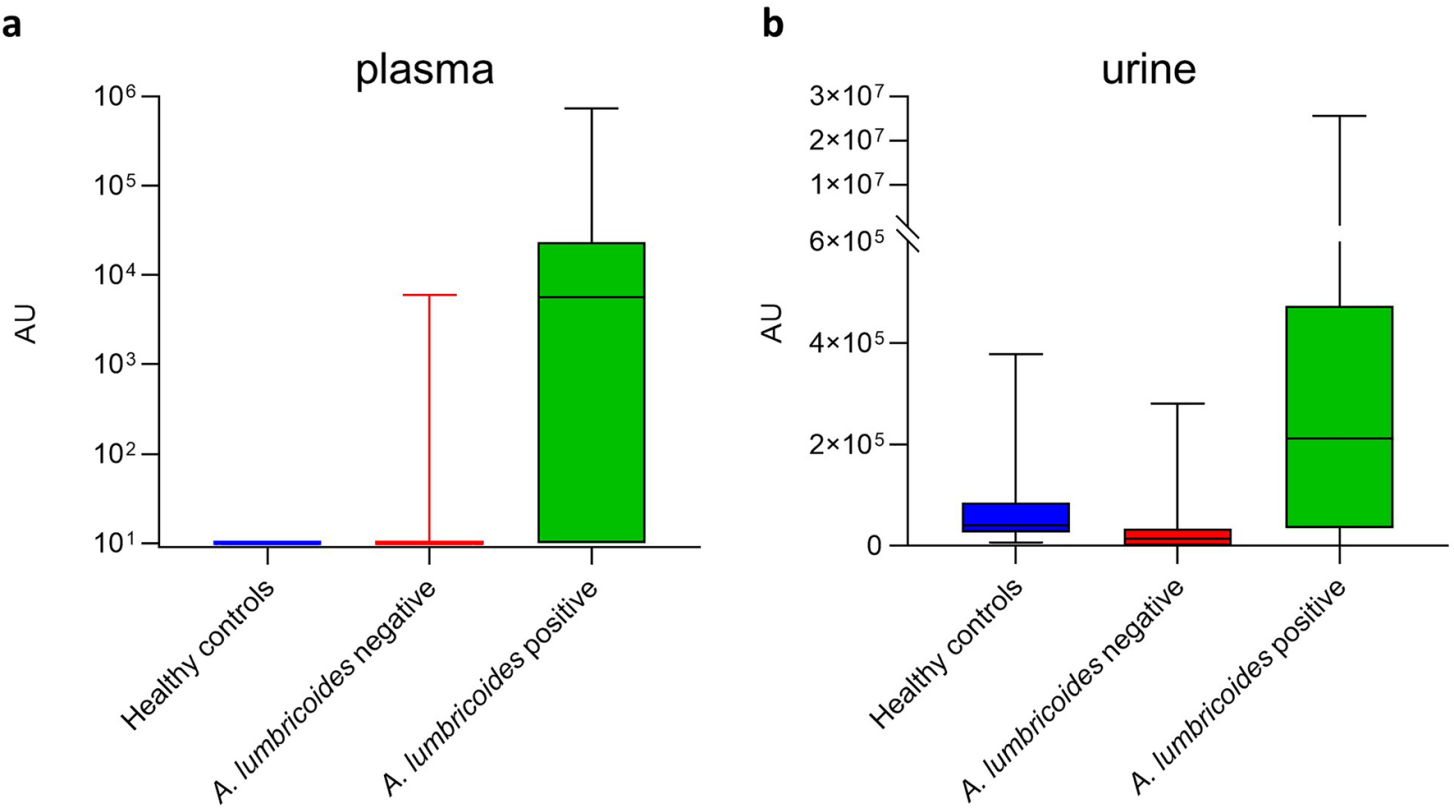
Discovery of an *Ascaris lumbricoides* specific biomarker. Box-and-whisker plots of the identified feature in plasma (**a**) and urine (**b**) of *A. lumbricoides*-positive (green, n = 38) subjects, endemic controls (red, n = 118) and healthy Belgian controls (blue, n = 40). AU = arbitrary units (intensity of ion current measured by the spectrometer).

**Figure 2:**
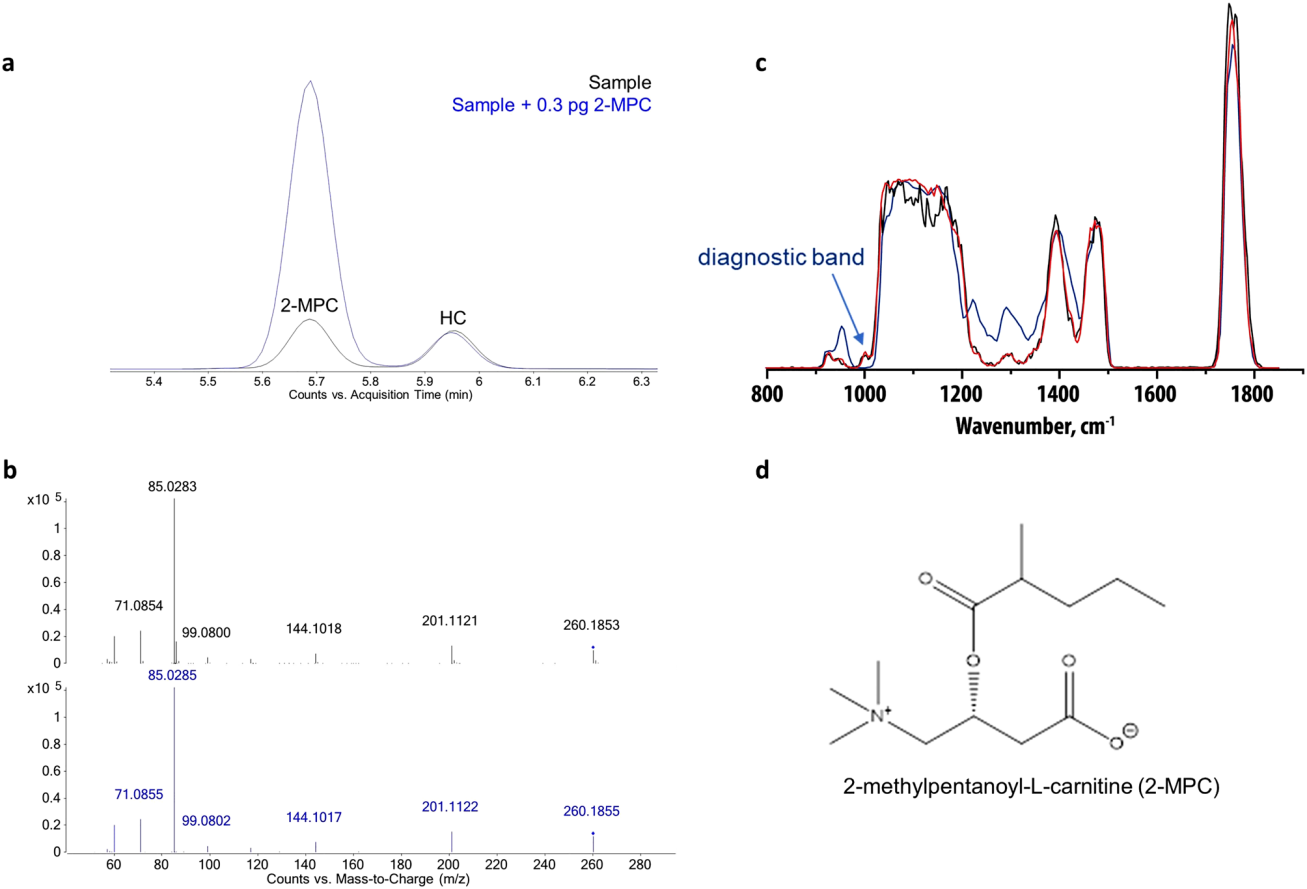
Structure confirmation of 2-methyl-pentanoyl-carnitine. (**a**) Extracted ion chromatograms of co-injection experiment of the synthetic 2-MPC spiked to a positive plasma extract (blue) in comparison to the positive plasma extract (black). (**b**) MS/MS fragmentation comparison of the synthetic and natural molecules (collision-induced dissociation at 20 V). (**c**) IR spectra generated with infrared ion spectroscopy (IRIS) analysis on the protonated ion at *m/z* 260 from the HPLC purified marker (black), 2-MPC (red) and 3-MPC (blue). (**d**) Structure of 2-MPC.

Sample extracts were analyzed by LC–MS, and statistical analysis (Mann–Whitney U test) identified several features with significant association to *A. lumbricoides* infection (Supplementary Table S1). One feature with an accurate mass of 259.178 Da and a retention time of 5.68 min was enriched in both plasma and urine from *A. lumbricoides*-positive subjects. The corresponding box-and-whisker plots for this feature indicated elevated signal intensities in infected patient samples (Fig. 1; *p* < 0.001). The most likely elemental composition, based on accurate mass measurements and isotope readout provided by high-resolution MS, corresponds to C_13_H_25_NO_4_. Based on MS/MS fragmentation analysis, this feature could tentatively be identified as hexanoyl carnitine. MS/MS spectra were compared to a synthetic standard of hexanoyl carnitine and seemed to match. Chromatographic behavior though suggested a slightly deviating (more hydrophilic) structure, viz a branched-chain stereoisomer n-methyl-pentanoyl-carnitine (Supplementary Fig. S1). To further confirm this structure, both 2-MPC and 3-MPC were generated via chemical synthesis (Supplementary Materials and Methods). Upon injection of both compounds, 2-MPC had the same retention time of the natural marker, hence confirming identity (Fig. 2a,b). Final proof of the structure was provided by the infrared spectra obtained with infrared ion spectroscopy (IRIS) analysis on ions generated from HPLC purified material and solutions of both synthetic materials (Fig. 2c and Supplementary Fig. S2), which showed the natural marker had exactly the same IR spectrum as 2-MPC (Fig. 2d).

After confirmation of the molecular structure of 2-MPC, we developed a targeted LC–MS/MS method using synthetic 2-MPC as reference material to construct calibration curves. This method was shown to be able to separate 2-MPC from 3-MPC with minimal overlap, but without baseline separation (Supplementary Fig. S3). Using this method, different sample sets from subjects from Kenya (n = 771), Indonesia (n = 220), Ethiopia (n = 60) and Belgium (n = 214) were evaluated, and two cut-offs were determined for 2-MPC as urinary marker for *A. lumbricoides* infection: (i) a low cut-off for detection of infection, and (ii) a high cut-off for detection of M&HI infection (causing morbidity).

**Figure 3:**
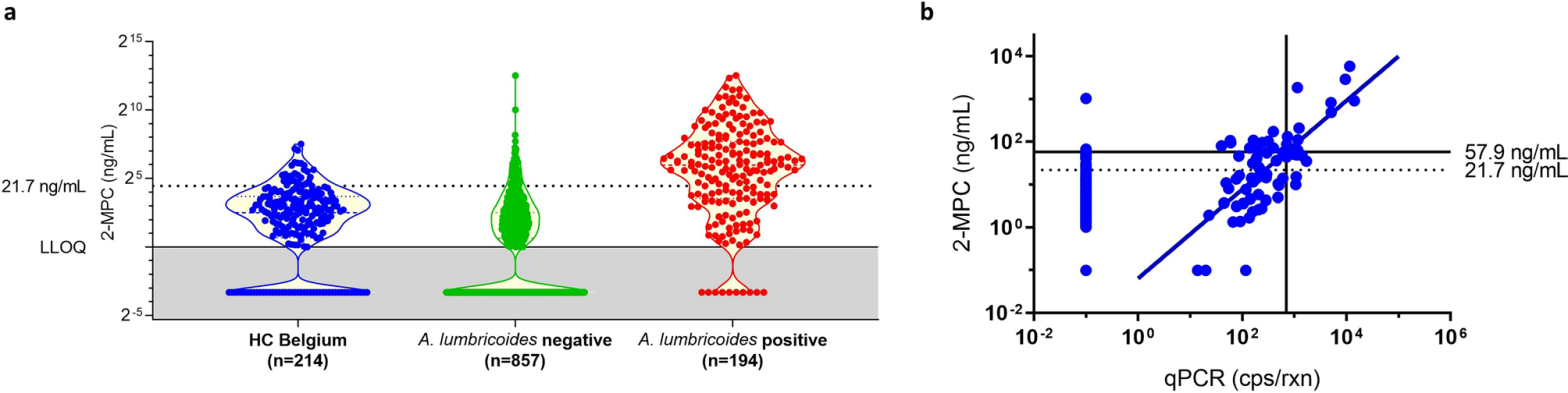
Determination of a low and high cut-off for detection of infection. (**a**) Quantification of urinary 2-MPC in *A. lumbricoides*-positive (red, n = 195) subjects, endemic controls (green, n = 858) and healthy Belgian controls (blue, n = 214). Grey area indicates samples that were below lower limit of quantification (1 ng/mL). A low cut-off to identify *A. lumbricoides*-infected subjects was determined using ROC analysis of *A. lumbricoides*-positive *vs.* healthy controls and found to be 21.7 ng/mL. (**b**) Correlation between urinary 2-MPC and *A. lumbricoides* DNA detection in stool collected in Kenya (expressed in *A. lumbricoides* copies/reaction) was used to determine a high cut-off of 57.9 ng/mL 2-MPC that could be used to identify subjects with moderate-to-high infection. Moderate infection was defined as > 700 cps/rxn (see “Materials and methods” section).

For all samples from STH-endemic countries, *A. lumbricoides, T. trichiura* and hookworm infections were determined based on either Kato-Katz thick smear (Ethiopia) or qPCR (Kenya and Indonesia). A ROC analysis was performed using all *A. lumbricoides* infected samples as positive group and all healthy controls from Belgium as negative group (Fig. 3a). The area under curve (AUC) was 0.81 and based on this ROC analysis, a cut-off for infection was determined at 21.7 ng/mL. In order to determine the high cut-off, the relationship between urinary 2-MPC levels and qPCR result in the Kenyan sample set was determined (Fig. 3b). Based on all samples that were found to be qPCR positive, a correlation was found between qPCR and urinary 2-MPC (slope = 1.038, r^2^ = 0.499, *p* < 0.0001). Based on this correlation, a cut-off for M&HI infection was determined at 57.9 ng/mL (corresponds to 700 cps/rxn, see “Materials and methods” section).

**Figure 4:**
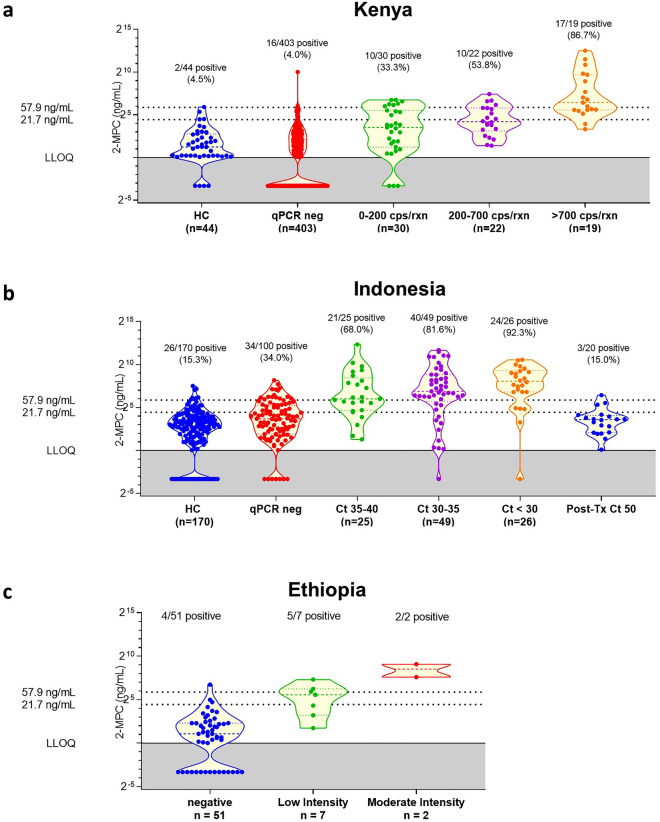
Violin plots of the quantitative values obtained in the different populations, binned according to infection intensity (either qPCR or Kato-Katz).

Based on these cut-offs, the obtained quantitative values for urinary 2-MPC in the different populations were further investigated in detail (Fig. 4). As anticipated, the data indicated an increased level of urinary 2-MPC upon increasing *A. lumbricoides* infection intensity, an observation that was seen in the sample sets from Kenya (Fig. 4a), Indonesia (Fig. 4b), and Ethiopia (Fig. 4c). There was however a significant overlap between different groups, as well as between uninfected and infected subjects. This was also reflected in the diagnostic parameters determined on these data (Table 2). Total accuracy was found to be 85.7% when using the low cut-off to determine infection and 90.5% when using the high cut-off to detect M&HI infections. However, sensitivity for both cut-offs was only 71.1% and 74.0%, respectively. Specificity on the other hand was high, with 90.2% and 92.9% for low and high cut-off, respectively.

**Table 2:** Diagnostic parameters of urinary 2-MPC as marker for *A. lumbricoides*.

	n	TP	TN	FP	FN	Se (%)	Sp (%)	Acc (%)	PPV (%)	NPV (%)
**Scenario 1: Low cut-off to identify infection**										
Kenya	474	37	387	16	34	52.1	96.0	89.5	69.8	91.9
Indonesia	220	85	83	37	15	85.0	69.2	76.4	69.7	84.7
Ethiopia	60	6	48	3	3	66.6	94.1	90.0	66.6	94.1
Total	754	128	518	56	52	71.1	90.2	85.7	69.6	90.9
**Scenario 2: High cut-off to identify moderate-to-high infection**										
Kenya	474	11	442	13	8	57.9	97.1	95.6	45.8	98.2
Indonesia^a^	220	58	115	30	17	77.3	79.3	78.6	65.9	87.1
Ethiopia	60	2	54	4	0	100	93.1	93.3	33.3	100
Total	754	71	611	47	25	74.0	92.9	90.5	60.2	96.1

^a^M&HI infection in Indonesian samples was defined as Ct < 35, based on^12^.

*TP* true positives, *TN* true negatives, *FP *false positives, *FN* false negatives, *SE* sensitivity, *SP* specificity, *Acc* accuracy, *PPV* positive predicted value, *NPV* negative predictive value.

In order to confirm that 2-MPC is uniquely associated to *A. lumbricoides* infection and that other STH (*T. trichiura* and hookworm) and/or *S. mansoni* infections are not associated to 2-MPC levels, the samples from Kenya, Indonesia and Ethiopia were grouped according to infection type and one-way ANOVA was performed on the 2-MPC data of the single infected subjects. These analyses demonstrated that there was no significant difference in 2-MPC levels between uninfected subjects and subjects infected with *T. trichiura*, hookworm or *S. mansoni* (*p* > 0.05), while *A. lumbricoides* infected subjects did differ highly significant from uninfected ones in each of the three countries evaluated (Table 3).

**Table 3:** One-way ANOVA based comparison of 2-MPC data between uninfected endemic subjects and subjects infected with *A. lumbricoides*, *T. trichiura*, hookworm or *S. mansoni,* respectively.

	*A. lumbricoides*	*T. trichiura*	Hookworm	*S. mansoni*	Uninfected
**Kenya**					
n	48	18	9	75	303
*p*-value	< 0.0001	> 0.999	> 0.999	0.3745	n.a
**Indonesia**					
n	50	50	50	0	20
*p*-value	< 0.0001	> 0.999	> 0.999	n.d	n.a
**Ethiopia**					
n	9^a^	25	0	2	24
*p*-value	0.0061	0.5964	n.d	0.7324	n.a

^a^Since only 4 out of a total of 9 *A. lumbricoides* infected subjects was single infected, the subjects co-infected with *T. trichiura* were also included here.

*n.a.* not applicable as this is the reference group, *n.d.* not determined.

**Figure 5:**
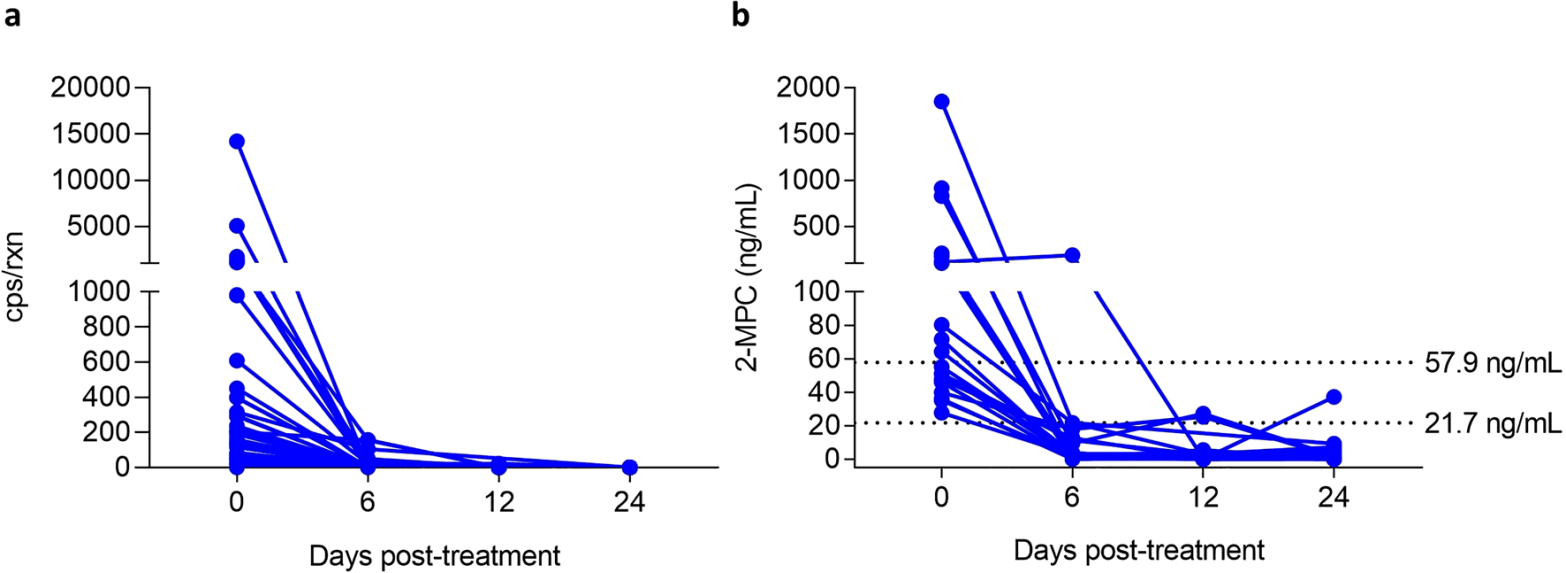
Effect of treatment with albendazole on the presence of *Ascaris lumbricoides* DNA in stool (**a**) and urinary 2-MPC (**b**). Stool and urine samples were collected before (Day 0) and at different timepoints after treatment with albendazole: 6 days, 12 days and 24 days after treatment.

To further establish the connection between 2-MPC in urine and *A. lumbricoides* infection, we analyzed urine samples from patients with *A. lumbricoides* infection who were treated with albendazole. Samples were collected at 0, 6, 12 and 24 days after treatment. qPCR analysis on concurrently collected stool samples showed complete disappearance of STH DNA in all subjects, indicating killing or expulsion of the parasite (Fig. 5a). The quantification of 2-MPC levels in the different urine samples revealed a strong reduction in 2-MPC levels, at all timepoints post-treatment, with only 1 out of 21 (4.8%) subjects having urinary 2-MPC level above the low cut-off at day 24 (Fig. 5b). These results further establish the value of 2-MPC as a means to monitor patent *A. lumbricoides* infection.

**Figure 6:**
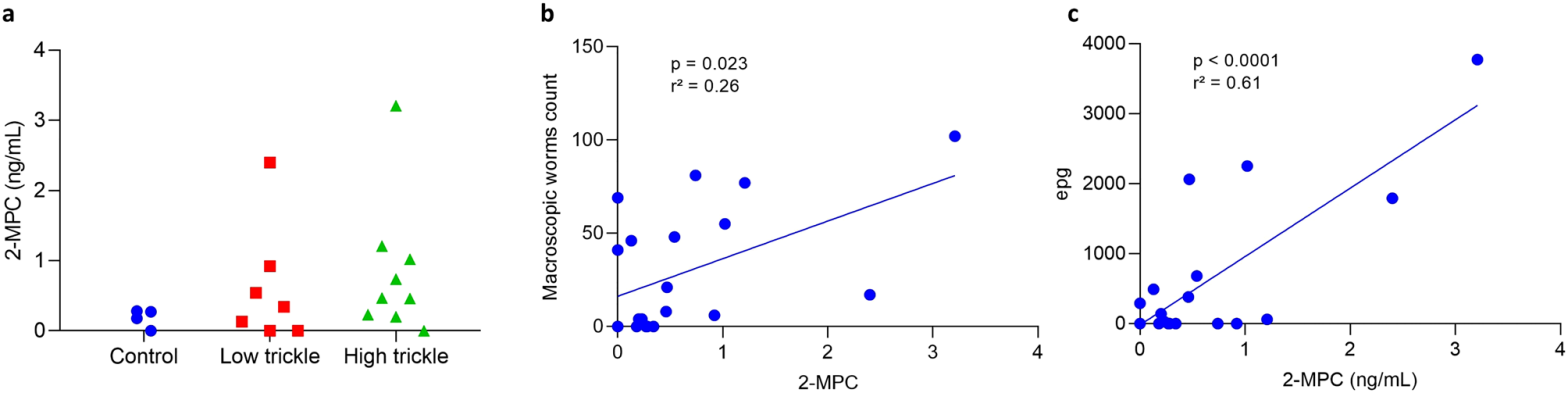
Quantification of urinary 2-MPC in *A. suum* infected pigs: control group, low and high trickle infected pigs (**a**) and correlation between urinary 2-MPC and macroscopic worms count (**b**) and epg count (**c**).

Since trickle infection of pigs with *A. suum* is a good model to study *A. lumbricoides* infection^13^, 2-MPC levels were determined in urine samples collected from 20 pigs 56 days post-infection: 4 pigs were uninfected (control), 7 pigs were experimentally inoculated with 20 *A. suum* eggs/day (low trickle) and 9 pigs were experimentally inoculated with 100 *A. suum* eggs/day (high trickle). Infection status was confirmed by determination of fecal egg counts (FECs) and worm counts in the intestines of necropsied animals (Table 4). Interestingly, none of the pig urine samples had 2-MPC levels above 4 ng/mL, being far below the cutoff proposed to determine infection in humans (21.7 ng/mL) (Fig. 6a). No statistically significant difference in 2-MPC levels was observed between the different groups, but a trend towards increased 2-MPC levels in infected pigs compared to control pigs could be observed. We therefore assessed whether the urinary 2-MPC levels were associated to other, more direct measures of infection. This analysis demonstrated a significant association between urinary 2-MPC levels and both macroscopic worm counts (*p* = 0.023), and FECs (*p* < 0.0001) (Fig. 6b,c). The latter observation might indicate that also in pigs, urine 2-MPC is a biomarker for *Ascaris* infection, but levels appear to be much lower compared to those found in human urine.

**Table 4:** Overview of infection status of pigs in the different infection groups. For each of the animals epg counts in stool and macroscopic worm counts in the intestines were determined.

Group	n	Fecal egg counts (epg; median; min–max)	Median worm counts (median; min–max)
Control	4	0 (0–0)	0 (0–0)
Low trickle	7	290 (0–1790)	41(0–69)
High trickle	9	140 (0–3770)	21 (0–102)

*epg* eggs per gram.

## Discussion

**Figure 7:**
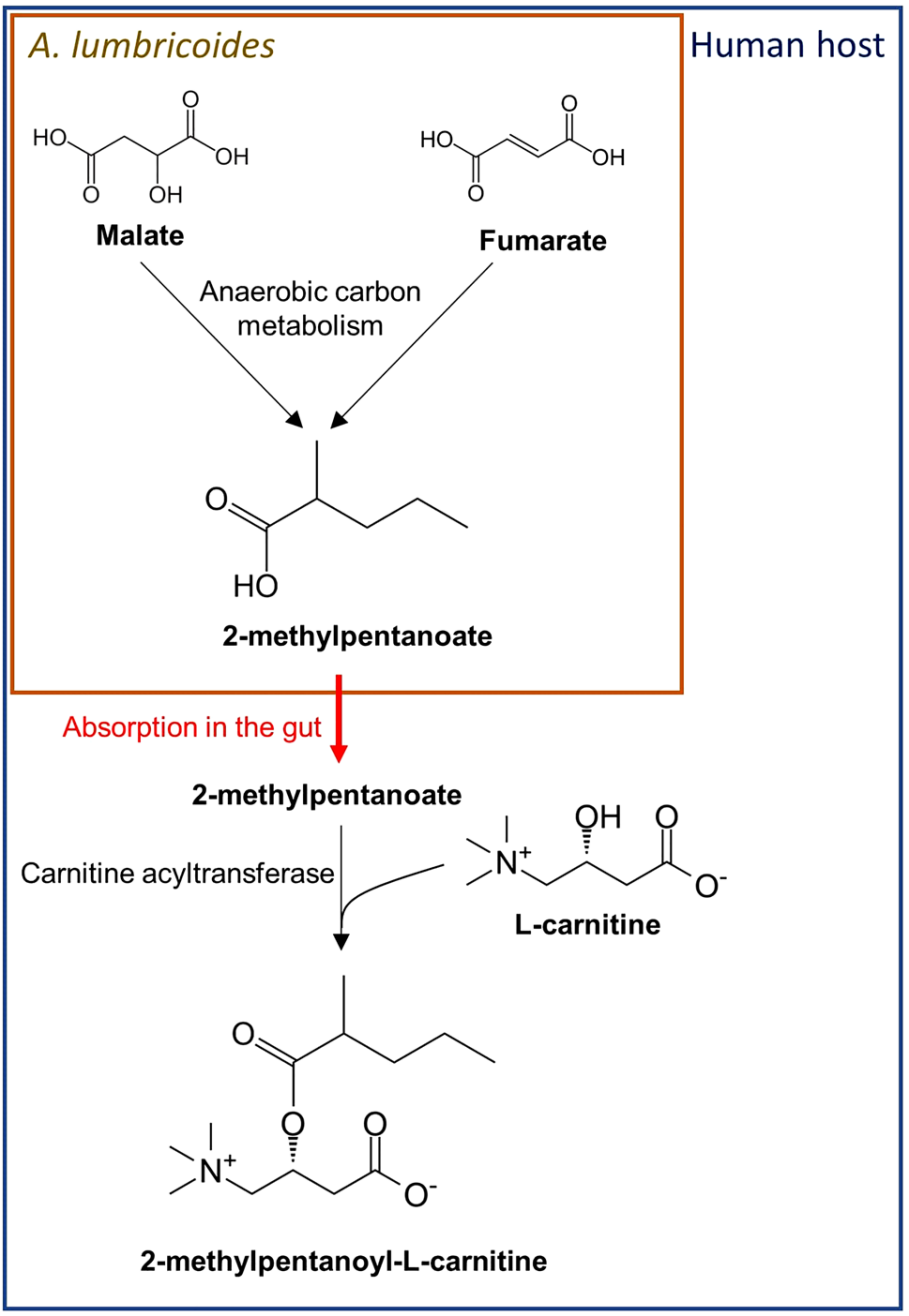
Presumable metabolic pathway of 2-MPC. The parasitic anaerobic carbon metabolism pathway leading to 2-methylpentanoate has been described in detail before^48^.

In this work, 2-MPC was identified as a potential biomarker for infection with *A. lumbricoides*. Although this metabolite was found to be significantly increased both in plasma and urine of infected individuals, we have only further explored the possible use of this metabolite as a urinary marker. The presumable metabolic pathway of 2-MPC suggests the involvement of both *A. lumbricoides* and its human host in the biosynthesis of this metabolite (Fig. 7). 2-MPC is a branched chain fatty acid ester of 2-methyl pentanoate and carnitine. One of the physiological roles of carnitine is to facilitate the excretion of fatty acids in urine, indicating that the excretion of 2-MPC is a way for the human host to excrete the fatty acid 2-methyl pentanoate^14^. Notedly, 2-methyl pentanoate has been described as one of the end-products of the carbohydrate metabolism of *Ascaris suum*, the pig variant of *A. lumbricoides*^15,16^. Taken together, this would suggest that 2-MPC is produced by the human host in order to remove 2-methyl pentanoate that had been released by the helminth and adsorbed in the blood through the gut wall.

To enable interpretation of the 2-MPC values measured in urine, two cut-offs were established: a lower cut-off for infection at 21.7 ng/mL and a higher cut-off for moderate infection at 57.9 ng/mL. The performance of this novel biomarker was determined in three independent sample sets collected in different countries (Kenya, Ethiopia and Indonesia). Total accuracy (i.e. probability of correctly determining the infection status based on 2-MPC, either positive or negative) was found to be 85.7% when using the low cut-off to determine infection and 87.2% when using the high cut-off to detect M&HI infections. Since the performance in Kenya and Ethiopia was similar to the one found in Indonesia, the likelihood of urinary 2-MPC concentrations being driven by environmental factors is very small. Also, in the different study populations tested, several samples from individuals with other helminth infections (*T. trichiura*, hookworm and/or *S. mansoni*) were included and these were not found to have an impact on the urinary 2-MPC concentrations, indicating that 2-MPC is a specific marker for patent *Ascaris* infection. For *S. stercoralis*, this assessment could not be performed in the current study and follow-up studies will be needed to determine the impact of *S. stercoralis* infection on urinary 2-MPC levels. Additionally, a correlation was observed between infection intensity, as measured by stool-based qPCR and urinary 2-MPC levels. Also, urinary 2-MPC levels were found to decrease strongly in *Ascaris* infected subjects that were treated with albendazole, thereby providing further evidence of the relationship between the presence of adult *Ascaris* in the gut and 2-MPC in urine. It must be noted that there is some overlap between 2-MPC levels in urine from healthy controls or *A. lumbricoides* negative subjects and infected subjects. This might be caused by the fact that some healthy controls—that were not tested for helminth infections – were truly infected by either traveling to endemic areas or by zoonotic infection^17,18^. Also, some individuals from endemic areas that were negative by qPCR might still carry a low worm burden with only adult male or pre-adult worms in their gut as these will not result in shedding of eggs and DNA in stool; or were simply not detected by qPCR, something that has been observed before^8,19^. For the samples collected post-treatment, the remaining 2-MPC levels in otherwise Kato-Katz negative individuals may be attributed to worms which survived treatment, but with reduced or cessated egg production (drug-induced embryostatis), a phenomenon already demonstrated for *Onchocerca volvulus* and *A. suum*^20,21^. Alternatively, the analytical method used to determine 2-MPC might be affected by some interferences (possibly the closely eluting 3-MPC, but also other unknown molecules might be interfering), present in varying small quantities, but with the same mass fragment ions as 2-MPC and co-eluting at similar retention time. The consequence of this might be that cut-offs determined in this study are overestimated, thereby negatively impacting sensitivity of this biomarker. But it can also not be excluded that 2-MPC is naturally present in urine of non-infected individuals and is caused by e.g., the presence of certain gut bacteria as it is known that they produce several of these short branched-chain fatty acids, mainly isobutyrate and isovalerate^22–24^.

Since trickle infection of pigs with *A. suum* is often used as a model to study the host-parasite relationship, we also examined 2-MPC concentrations in urine samples of pigs infected with *A. suum*. Although absolute levels were much lower than those found in humans, we did observe a correlation between urinary 2-MPC levels and both FECs and worm counts. This might indicate that also in pigs urinary 2-MPC is a biomarker for *Ascaris* infection. Why 2-MPC levels were lower than those found in human urine is not clear, but a difference in host metabolism might play a role. Of interest, total plasma carnitine levels in humans were found to be on average around 45 µM, while in pigs these levels were typically at least 3 times lower^25–28^. But it could also be possible that pigs have a different mechanism of clearing branched-chain fatty acids from their system.

Although the data presented here suggest a direct connection between the presence of *A. lumbricoides* and 2-MPC in urine, it cannot be excluded that 2-methyl valeric acid (and consequently, its carnitine ester 2-MPC) is in fact produced by gut bacteria. There are several reports that indicate intestinal parasites, such as *A. lumbricoides*, increase host gut microbiome diversity^29–33^. This interplay between host, microbiome and macrobiome might lead to an altered production of certain short-chain fatty acids (SCFAs) by the microbiome. In this respect, increased levels of gut microbiota derived SCFAs were found in feces of pigs chronically infected with *A. suum*^34^*.* It must however be said that these changes are rather small compared to the large (log-scale) changes observed in the study presented here. Also, our observation that 2-MPC is specific for *Ascaris* infection would disfavor the hypothesis of the increased bacterial production of this specific SCFA as the increased SCFA production was observed across diverse helminth species^34^. Furthermore, the correlation between *A. lumbricoides* infection intensity and the urinary 2-MPC levels suggest a rather direct link between the worm and 2-MPC levels as it is unlikely that increased helminth infection would lead to an equally increased level of microbiota responsible for the production of 2-methyl valeric acid. Lastly, the fact that albendazole has such a rapid and dramatic impact on the 2-MPC levels found in *A. lumbricoides* infected subjects also suggest 2-MPC is directly connected to the helminth infection as it was shown before that albendazole treatment in *A. lumbricoides* infected subjects had significant impact on microbiome composition 3 months post-treatment but almost no impact after 3 weeks^35^.

Previous reports have suggested that 2-methyl butyramide and 2-methyl valeramide or even the corresponding acids (2-methyl butyric acid and 2-methyl valeric acid) could be considered urinary biomarkers for active *A. lumbricoides* infection^10,36^. Similar as observed here for 2-MPC, there seemed to be a correlation between metabolite concentration and worm burden. However, more recent investigation of these proposed biomarkers could not confirm this and in fact has suggested that the original metabolite identification might have been incorrect^11^. Whether (one of) the biomarkers originally detected correspond to 2-MPC is hard to prove but given the similarities in the chemical structures between 2-methyl valeramide and 2-MPC, it could be possible that the same marker was detected but identified as another chemical structure due to differences in analytical techniques available at that time.

In summary, we have identified a urinary metabolite 2-MPC that is thought to be a human metabolite of 2-methyl pentanoic acid, one of the end products of *A. lumbricoides* carbon metabolism^15,16^. The correlation of this marker with infection intensity and the fact that albendazole treatment results in greatly reduced concentrations suggest an intimate relationship between this novel biomarker and the infection with adult *A. lumbricoides*. The discovery of 2-MPC as biomarker for *A. lumbricoides* infection could lead to the development of novel diagnostic tools to monitor infection. Further investigation of the clinical utility of urinary 2-MPC, in either high or low-transmission setting will be required to that end.

## Materials and methods

### Human study samples

Plasma and urine samples from Kenya were collected as part of a field study. The study was approved by the KEMRI Scientific and Ethics Review Unit (SERU), Nairobi, Kenya (Protocol Nr. # KEMRI/SERU/CGHR/102/3554). Since all study participants were minors, informed consent forms were signed by parents/guardians of the study participants, and verbal assents were obtained from all study participants. This study was undertaken in the former Nyanza province, in the southwest part of Kenya, with collections in the Kisumu county (high *S. mansoni* prevalence area) and Siaya county (high STH prevalence area). Stool samples were collected in order to determine the STH and *S. mansoni* infection status, based on both microscopic egg counting by the Kato-Katz thick smear and qPCR-based quantification of helminth DNA present in stool. qPCR data were chosen as the reference data set due to the higher sensitivity of qPCR compared to Kato-Katz^8^. A total of 476 subjects were included in this study, of which 474 had donated plasma samples, 475 had donated stool and all 476 had donated urine samples. An overview of the study population, including parasitological information is provided in Supplementary Table S2. After the cross-sectional collection of samples, a total of 105 individuals were treated with a single oral dose of albendazole (400 mg) and urine and stool samples were collected at day 6, day 12 and day 24 after treatment. An overview of the parasitological information of these individuals on the different visits is provided in Supplementary Table S3.

Additionally, a set of 220 urine samples from individuals with or without STH infection, collected in Flores, Indonesia was kindly provided by Maria Yazdanbakhsh (Leiden University Medical Center, Leiden, The Netherlands)^12^. These samples were from a study that has been approved by the ethical committee of Faculty of Medicine Universitas Indonesia (ref: 549/H2.F1/ETIK/2013), and has been filed by the ethics committee of Leiden University Medical Center, clinical trial number: ISRCTN75636394. The study, its benefits and risks were explained to the population and consent forms were distributed to be signed by the subjects who were willing to participate in this study. They were informed that they can withdraw from the study at any time, for any reasons and without any consequences.

We also included a collection of urine samples from 60 individuals infected with STHs and/or *S. mansoni*, collected in Jimma, Ethiopia. These samples were from a study that was reviewed and approved by the Ethical Review Board of Jimma University, Jimma, Ethiopia: IHRPGD/469/18 & IHRPGD/681/2019. Parent(s)/guardians of participants signed an informed consent document indicating that they understood the purpose and procedures of the study, and that they allowed their child to participate. If the child was ≥ 5 years, he or she had to orally assent in order to participate. Participants of ≥ 12 years of age were only included if they signed an informed consent document indicating that they understood the purpose and the procedures of the study and were willing to participate.

As a non-endemic control group, plasma and urine samples from Belgian healthy controls were included^37–41^. For all sample collections written informed consent was obtained from all individuals, and all samples were decoded and de-identified before they were provided for research purposes.

A detailed list of all study populations from endemic areas, including parasitological data is provided in Supplementary Table S4. *S. stercoralis* infection status was only available for samples from Indonesia, with all of them being negative. For samples from Kenya and Ethiopia, this was not assessed. All experiments were performed in accordance with relevant guidelines and regulations.

### Pig study samples

Twenty-five female and castrated male Rattlerow Seghers hybrid pigs were used for the infection experiment. Three groups of pigs were housed in separate, indoor pens in a helminth-free environment. All pigs had free access to a commercial feed and water. A first group of 5 pigs served as uninfected controls. A second group of 10 pigs received a trickle infection of 20 infective *A. suum* eggs for a total of 3 times per week. A third group of 10 pigs received a dose of 100 infective *A. suum* eggs three times per week. Doses were administered orally in a food bolus to each pig individually in order to mimic a low natural exposure. Pigs received infection doses for a total of 6 weeks. In total this corresponded with 360 or 1800 infective eggs given to each pig over the course of the trial depending on their respective infection group. On day 55, the number of *A. suum* eggs per gram of feces was determined for each pig using the Mini-FLOTAC method^42^. On the final day of the trial (Day 56), a urine sample was collected from 4 control pigs, 7 pigs in the low trickle group and 9 pigs of the high trickle group. We were unable to collect urine from some animals because they did not urinate at the time of sampling and the bladder was empty during necropsy. During necropsy, the content of the small intestine of each pig was sieved and the number of larval and adult *A. suum* worms counted.

### Stool based assessment of helminth infection

STH infections were assessed on stool samples using the Kato-Katz procedure and/or qPCR analysis, as described before^8,43^. For one sample, no qPCR result could be obtained, and this subject was excluded for further analysis, as well as the subject for which no stool sample was available. To determine a qPCR-based cut-off for M&HI infection, linear regression analysis was performed on log-transformed qPCR data (in copies per reaction (cps/rxn)) and Kato-Katz data (in eggs per gram (epg), Supplementary Fig. S4). Based on this analysis a qPCR result of 700 cps/rxn was found to correspond to 5000 epg, i.e. the boundary between low and moderate infection intensity^44^.

### Sample preparation and analysis for LC–MS based metabolomics

All sample preparation procedures, as well as all sample analyses used in the metabolomics based discovery of biomarkers for *A. lumbricoides* infection are described in Supplementary Materials and Methods^45^.

### Reference materials

Hexanoyl carnitine was purchased from Sigma-Aldrich (St. Louis, MO). The branched chain fatty acid carnitine esters 2-methyl pentanoyl carnitine (2-MPC) and 3-methyl pentanoyl carnitine (3-MPC) were synthesized in-house. A detailed description of the synthesis and quality control procedures is available in Supplementary Materials and Methods.

### Infrared ion spectroscopy (IRIS) analysis

A urine sample with high abundance of the feature of interest was used for collection. The peak corresponding to this feature was collected using an Agilent Technologies 1290 Infinity II series UHPLC equipped with an Acquity UPLC HSS T3 column and a fraction collector. A total volume of 250 µL purified product was collected and purity of the collection was confirmed by injection of a small volume onto the LC–MS method demonstrating the absence of any other isomeric peak in that collection. Both the compound purified from urine and the synthetic 2-MPC and 3-MPC references were subjected to infrared ion spectroscopy (IRIS) analysis using a modified Bruker AmaZon Speed ETD ion trap mass spectrometer and the FELIX IR free electron laser^46,47^. For each of the three compounds, protonated ions (*m/z* 260) were generated, mass-isolated and irradiated with FELIX. Resonant absorption of IR radiation increases the internal energy of the ions and induces fragmentation. A series of mass spectra is recorded with the IR laser frequency stepping through the spectral range of interest. IR spectra are reconstructed by plotting the dissociation yield (= ΣI(fragment ions)/(parent + ΣI fragment ions)) as a function of IR frequency. A detailed description of the IRIS experiments is provided in Supplementary Materials and Methods.

### LC high-resolution MS/MS (LC-HRMS/MS) based quantitation of 2-MPC in urine

After identification of 2-MPC in the metabolomics study, a targeted LC–MS/MS method was developed for analysis of 2-MPC in urine. The frozen urine samples were thawed on ice and centrifuged at 20,627 × g for 10 min. A volume of 100 µL of the samples was transferred, undiluted, to a vial with glass insert for LC–MS analysis.

The samples (2 µL injection volume) were injected on a Nexera UPLC system (Shimadzu, Kyoto, Japan) fitted with an Acquity UPLC HSS T3 column (2.1 × 100 mm; 1.8 μm particles; Waters, Milford, MA, USA) for separation. The column temperature was maintained at 50 °C. Elution was performed using an aqueous acidified (A: 0.1% formic acid in H_2_O) acetonitrile (B). Isocratic elution was achieved with 90% buffer A at a flow rate of 0.5 mL/min for 6 min. Subsequently, the percentage of buffer B was increased to 90% where it was maintained for 1 min before returning to the starting conditions (10% B) which were held for 1 min.

High-resolution accurate mass spectra were obtained with a TripleTOF 6600 Q-TOF mass spectrometer (AB Sciex, Ontario, Canada) equipped with a Turbo V electrospray ionization (ESI) source. The instrument was operated in electrospray ionization in the positive ion mode. Ion spray voltage was set to 5.5 kV, source temperature was set to 550ºC. The nebulizer gas was set to 50 psi and the heater gas to 55 psi, the de-clustering potential to 90 V.

Data were collected in both QTOF-MS (*m/z* 50–700, collision energy 10 V, accumulation time 50 ms) and SWATH-MS/MS (collision energy 30 V with a spread of 25 V, de-clustering potential of 70 V) experiments. During MS/MS experiments precursors of interest were included in an inclusion list. The instrument operated at high resolution mode (~ 30,000 FWHM) for TOFMS scans and high sensitivity mode (~ 15,000 FWHM) for TOFMS/MS scans. Mass calibrations were performed using positive ESI calibration solution (AB Sciex), which was automatically delivered by a calibrant delivery system (AB Sciex). Data acquisition was performed using the Analyst TF 1.7.1 software (AB Sciex). Quantitative data were obtained using Multiquant (v. 3.0.2). Individual compounds were quantified using the peak areas of the selected fragment ion from TOFMS/MS scans (*m/z* 85.028).

Calibration curves were prepared by spiking different amounts of synthetic 2-MPC to blank urine to obtain final concentrations between 0.1 and 20,000 ng/mL (resulting in a total of 17 calibration samples). Additionally, a blank sample was included. Endogenous linear regression (a regression model specifically designed for endogenous compounds) was performed using a 1/x^2^ weighting factor. Correlation coefficient of calibration curves was > 0.95. LLOQ was determined as the lowest concentration which could reliably be analyzed. In practice this comes down to the calibration point giving a signal-to-noise (S/N) ratio larger than 5. However, if the injection of a blank sample, after the injection of the highest calibrator, shows a higher S/N, the calibration point directly above this latter S/N ratio is taken as LLOQ.

### Statistical analysis

Statistical analyses used to analyze the data obtained in the untargeted discovery studies, have been described in Supplementary Materials and Methods. Receiver Operating Characteristics (ROC) analysis was performed using specified sample sets as cases and controls, and cutoff was determined as the point with maximal Youden’s index ((sensitivity + specificity) − 1). For evaluation of the correlation between urinary 2-MPC (in ng/mL) and qPCR-based detection of *A. lumbricoides* DNA in stool (in cps/rxn), linear regression analysis was performed on log-transformed data of positive samples only. For comparison of different infection groups, one-way ANOVA was performed assuming a non-Gaussian distribution (Kruskal–Wallis analysis). For determination of the diagnostic performance of 2-MPC, contingency tables were prepared using qPCR/Kato-Katz (as indicated) as the reference test and following parameters were determined: true positives (TP), true negatives (TN), false positives (FP) and false negatives (FN). Accuracy is defined as (TP + TN)/(TP + TN + FP + FN), sensitivity as TP/(TP + FN) and specificity as TN/(TN + FP). To assess correlation between 2-MPC in urine from pigs and FECs or worm counts, Spearman’s r correlation coefficient was determined. All analyses were performed using GraphPad Prism version 7.00.

### Ethics approval and consent to participate

Human samples from Kenya were collected as part of a field study in Kenya. The study was approved by the KEMRI Scientific and Ethics Review Unit (SERU), Nairobi, Kenya (Protocol # KEMRI/SERU/CGHR/102/3554). Since all study participants were minors, informed consent forms were signed by parents/guardians of the study participants, and verbal assents were obtained from all study participants.

Human samples from Indonesia used in this study are from a study that has been approved by the ethical committee of Faculty of Medicine Universitas Indonesia (ref: 549/H2.F1/ETIK/2013), and has been filed by the ethics committee of Leiden University Medical Center, clinical trial number: ISRCTN75636394. The study, its benefits and risks were explained to the population and consent forms were distributed to be signed by the subjects who were willing to participate in this study. They were informed that they can withdraw from the study at any time, for any reasons and without any consequences.

Human samples from Ethiopia are from a study that has been reviewed and approved by the Institutional Review Board (IRB) the Ethical Review Board of Jimma University, Jimma, Ethiopia: IHRPGD/469/18 & IHRPGD/681/2019. Parent(s)/guardians of participants signed an informed consent document indicating that they understood the purpose and procedures of the study, and that they allowed their child to participate. If the child was ≥ 5 years, he or she had to orally assent in order to participate. Participants of ≥ 12 years of age were only included if they signed an informed consent document indicating that they understood the purpose and the procedures of the study and were willing to participate.

All animal experiments were conducted in accordance with the E.U. Animal Welfare Directives and VICH Guidelines for Good Clinical Practice. Ethical approval to conduct the studies was obtained from the Ethical Committee of the Faculty of Veterinary Medicine, Ghent University.

### Consent for publication

Not applicable.

## Supplementary information

Supplementary information 1.

Supplementary information 2.

## Data Availability

All data generated or analyzed during this study are included in this published article [and its supplementary information files].

## Abbreviations

STHs: Soil-transmitted helminths
2-MPC: 2-Methyl pentanoylcarnitine
*A. lumbricoides*: *Ascaris lumbricoides*
*T. Trichiura*: *Trichuris trichiura*
LC–MS: Liquid chromatography coupled to mass spectrometry
HC: Hexanoyl carnitine
