# Supplementary material for: 2-Methyl-pentanoyl-carnitine (2-MPC): a urine biomarker for patent Ascaris lumbricoides infection
Source: Sci Rep. 2020 Sep 25;10:15780. doi: 10.1038/s41598-020-72804-y (PMC7519643; doi:10.1038/s41598-020-72804-y)
Supplement: Supplementary file 1 — Supplementary information 1. [file 41598_2020_72804_MOESM1_ESM.pdf]

2-methyl-pentanoyl-carnitine (2-MPC): a urine biomarker for patent *Ascaris lumbricoides* infection

Ole Lagatie[1*], Ann Verheyen[1], Stijn Van Asten[2], Maurice R. Odiere[3], Yenny Djuardi[4], Bruno Levecke[5], Johnny Vlaminck[5], Zeleke Mekonnen[6], Daniel Dana Medebo[6], Ruben T'Kindt[7], Koen Sandra[7], Rianne van Outersterp[8], Jos Oomens[8], Ronghui Lin[9], Lieve Dillen[2], Rob Vreeken[2], Filip Cuyckens[2] and Lieven J. Stuyver[1]

[1] Janssen Global Public Health, Janssen R&D, Turnhoutseweg 30, 2340 Beerse, Belgium

[2] Discovery Sciences, Janssen R&D, Turnhoutseweg 30, 2340 Beerse, Belgium

[3] Kenya Medical Research Institute, Centre for Global Health Research, P. O. Box 1578, 40100 Kisumu, Kenya

[4] Department of Parasitology, Faculty of Medicine, Universitas Indonesia, Jakarta, Indonesia

[5] Department of Virology, Parasitology and Immunology, Ghent University, Salisburylaan 133, 9820 Merelbeke, Belgium

[6] School of Medical Laboratory Sciences, Jimma University, Jimma, Ethiopia

[7] Research Institute for Chromatography, President Kennedypark 26, 8500 Kortrijk, Belgium

[8] FELIX Laboratory, Faculty of Science, Radboud University, Toernooiveld 7, 6525 ED Nijmegen, The Netherlands

[9] Janssen R&D, Welsh & McKean Road, Spring House, Pennsylvania, 19477-0776, USA

[*] Corresponding author

Table S1. Features identified in untargeted LC-MS to be associated with *A. lumbricoides* infection

Matrix	ESI	RT (min)	Mass	Formula	Name	p_{corr}	FC
Plasma	-	4.512	356.2040	C15 H32 O9	glycerol polymer	< 0.001	*Inf.*
Plasma	-	4.769	176.9924	C5 H7 N O2 S2	unknown	< 0.001	*Inf.*
Plasma	-	5.761	420.1759	C15 H31 Cl O8	disinfection by-products (DBPs) in drinking water (formic acid adduct)	< 0.001	*Inf.*
Plasma	-	5.763	448.2069	C18 H37 Cl O10	disinfection by-products (DBPs) in drinking water	< 0.001	*Inf.*
Plasma	-	5.770	209.0690	C10 H11 N O4	unknown	< 0.001	2.24
Plasma	-	5.848	420.1760	C15 H31 Cl O8	disinfection by-products (DBPs) in drinking water (formic acid adduct)	< 0.001	*Inf.*
Plasma	-	6.940	235.0840	C12 H13 N O4	unknown	0.048	*Inf.*
Plasma	-	7.860	412.1650	C21 H28 O6 (formate)	unknown	0.001	*Inf.*
Plasma	-	7.930	180.0780	C10 H12 O3	unknown	0.036	*Inf.*
Plasma	-	8.760	221.9480	C7 H4 Cl2 O4	unknown	< 0.001	1.92
Plasma	-	9.940	178.0630	C10 H10 O3	unknown	< 0.001	*Inf.*
Plasma	+	4.329	282.1678	C12 H26 O7	unknown	< 0.001	*Inf.*
Plasma	+	4.503	356.2040	C15 H32 O9	glycerol polymer	< 0.001	*Inf.*
Plasma	+	5.674	259.1772	C13 H25 N O4	2-methylpentanoylcarnitine	< 0.001	*Inf.*
Plasma	+	5.753	448.2068	C18 H37 Cl O10	disinfection by-products (DBPs) in drinking water	< 0.001	*Inf.*
Plasma	+	5.753	374.1704	C15 H31 Cl O8	disinfection by-products (DBPs) in drinking water	< 0.001	*Inf.*
Plasma	+	5.847	374.1705	C15 H31 Cl O8	disinfection by-products (DBPs) in drinking water	< 0.001	*Inf.*
Plasma	+	6.065	241.1456	C16 H19 N O	unknown	0.002	*Inf.*
Urine	-	5.430	372.1080	C16 H20 O10	C10H12O4 - glucuronide	0.005	*Inf.*
Urine	-	5.780	209.0690	C10 H11 N O4	unknown	0.003	5.82
Urine	-	6.100	196.0720	C10 H12 O4	unknown	< 0.001	*Inf.*
Urine	+	4.70	174.0710	C8 H14 O2 S	C8H14O2S	0.001	*Inf.*
Urine	+	5.68	259.1780	C13 H25 N O4	2-methylpentanoylcarnitine	0.001	3.27
Urine	+	6.55	538.2050	C26 H34 O12	unknown	0.033	*Inf.*
Urine	+	6.68	386.1220	C17 H22 O10	C11H14O4 - glucuronide	0.009	*Inf.*
Urine	+	6.79	386.1210	C17 H22 O10	C11H14O4 - glucuronide	0.005	*Inf.*
Urine	+	6.95	235.0840	C12 H13 N O4	C10H11O3 - glycine	< 0.001	3.33
Urine	+	7.32	265.0960	C13 H15 N O5	C11H12O4 - glycine	0.009	*Inf.*
Urine	+	8.22	254.1150	C13 H18 O5	unknown	< 0.001	4.47

Table S2. List of samples used for performance evaluation of 2-MPC as a biomarker for *A. lumbricoides* infection

**Origin**	**Kenya**	**Indonesia**	**Ethiopia**	**Belgium**
Total (n)	476	220	60	214
A. lumbricoides pos (n)	71	100	9	n.d.
T. trichiuris pos (n)	30	75	30	n.d.
Hookworm pos (n)	16	75	2	n.d.
S. mansoni pos (n)	82	n.d.	3	n.d.

Table S3. List of samples from albendazole treated individuals from Kenya

**Visit**	Screening	Visit 1 (6 days post treatment)	Visit 2 (12 days post treatment)	Visit 3 (24 days post treatment)
Total (n)	105	105	93	103
A. lumbricoides pos (n)	38	25	2	0
T. trichiuris pos (n)	46	38	35	42
Hookworm pos (n)	5	2	2	4
S. mansoni pos (n)	11	6	6	9

Suppl. Figures

Fig. S1. Co-injection experiment hexanoyl carnitine

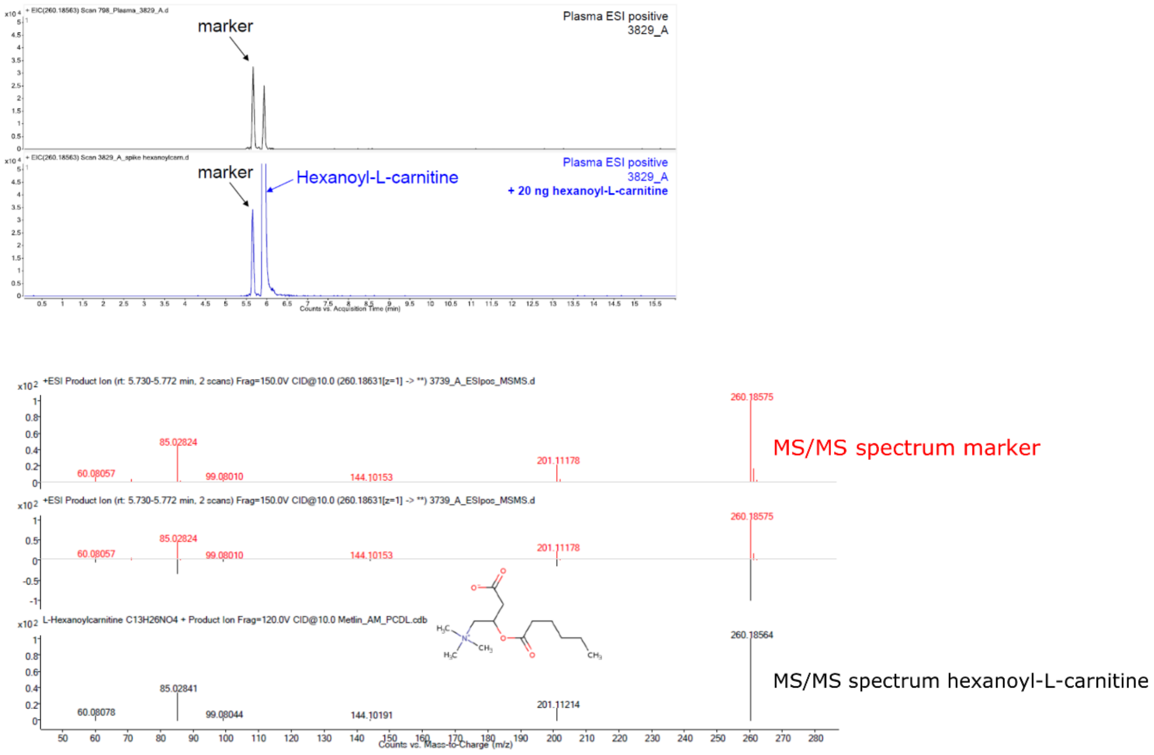

Fig. S2. IR spectra generated with infrared ion spectroscopy (IRIS) analysis on the collision-induced fragment at *m/z* of 201for the HPLC purified marker (black), 2-MPC (red) and 3-MPC (blue)

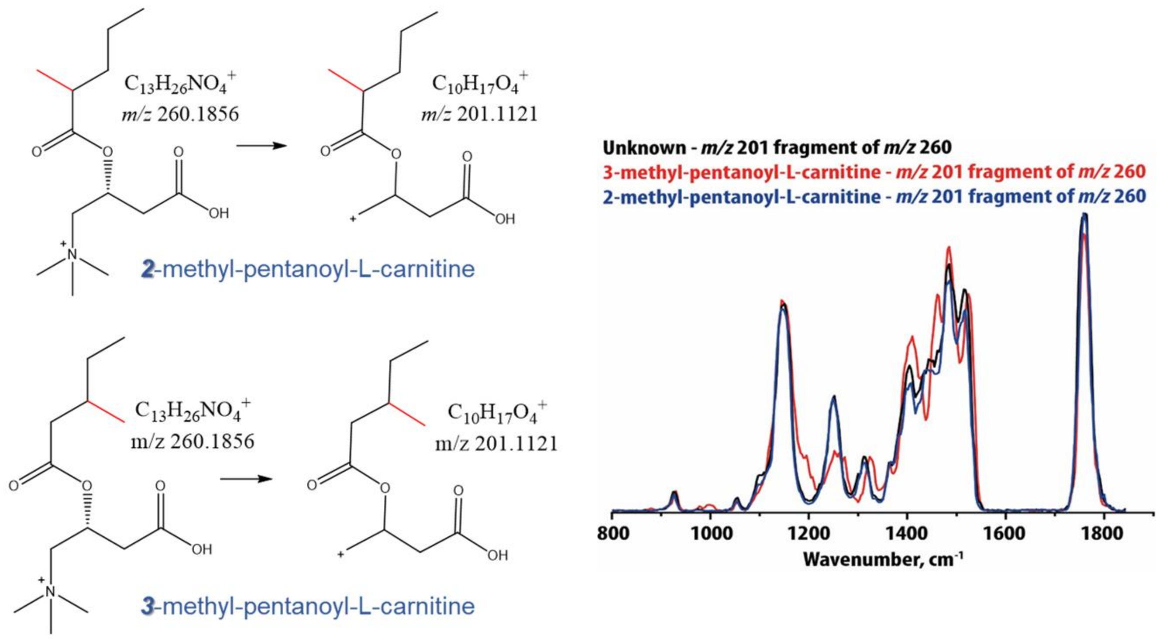

Fig. S3. Extracted ion chromatograms of synthetic 2-MPC, 3-MPC and a mixture of both synthetic compounds.

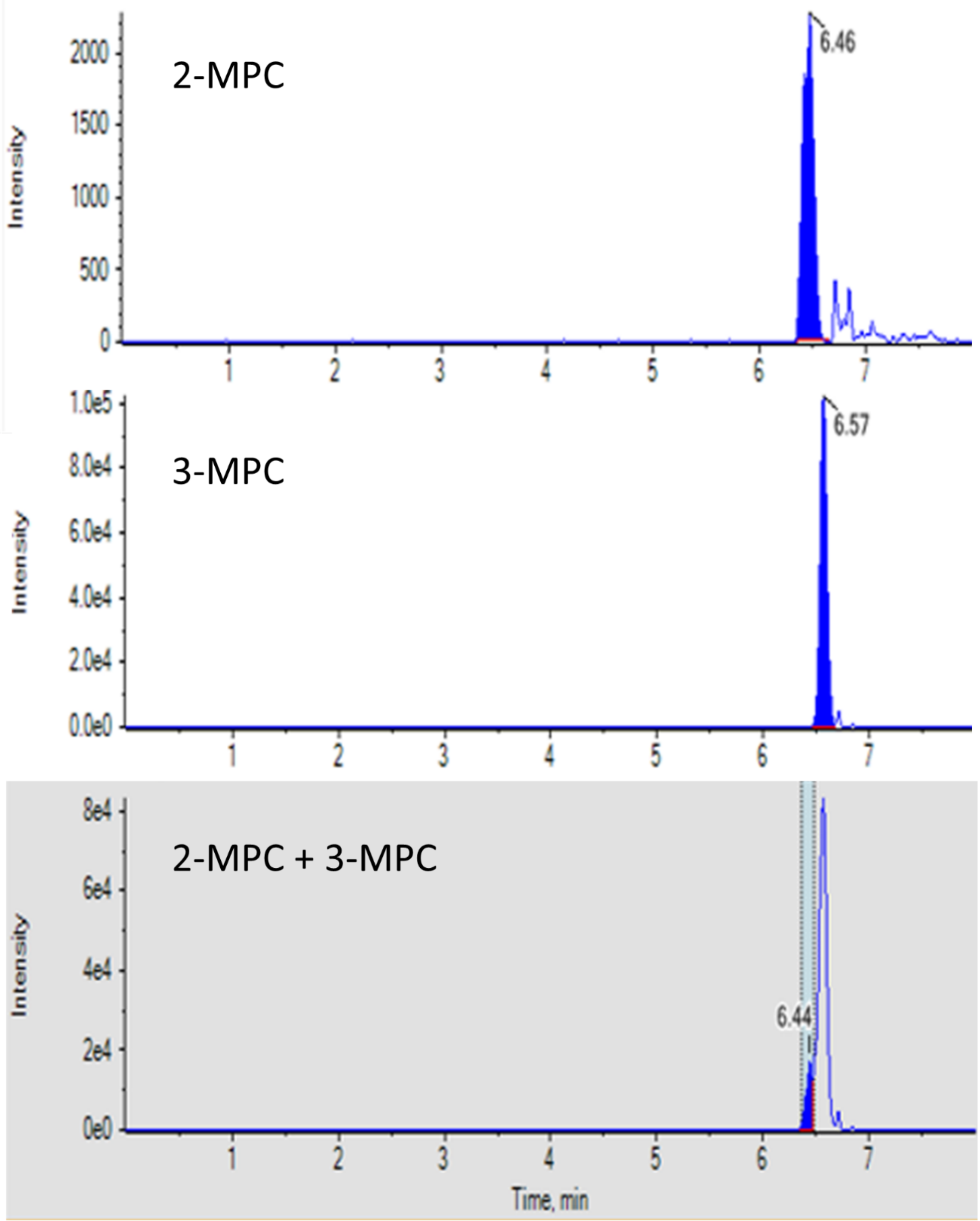

Fig. S4. Correlation between *A. lumbricoides* FECs (expressed in epg) and *A. lumbricoides* DNA detection in stool collected in Kenya (expressed in *A. lumbricoides* copies/reaction) was used to determine a cut-off of 700 copies/reaction that could be used to identify subjects with moderate-to-high infection.

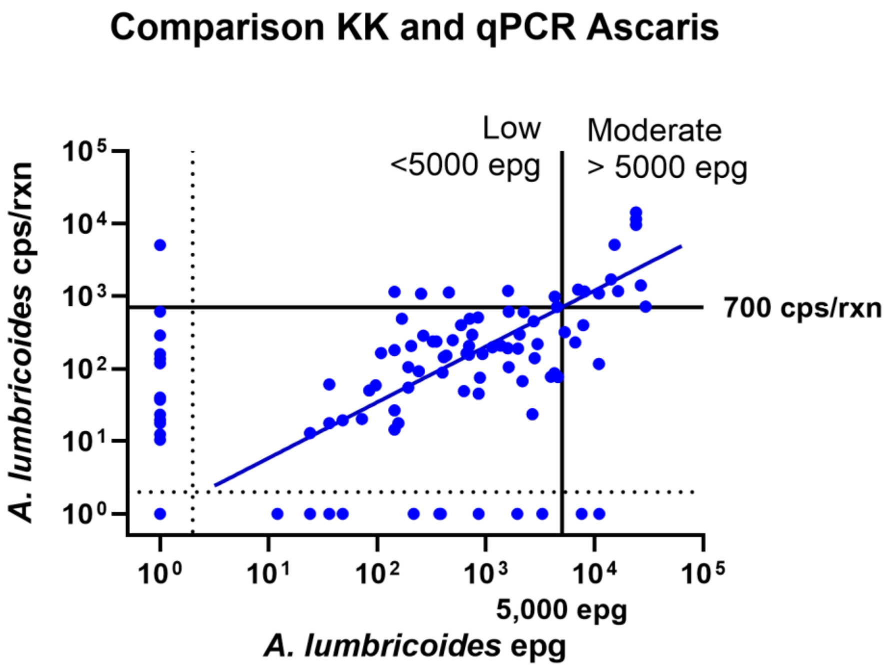

Suppl. Material and methods

Preparation of QC samples for metabolomics

A quality control (QC) pool was constructed by collecting 50 or 100 µL of all the plasma or urine samples, respectively, that were used for the untargeted discovery approaches. Subsequently, this QC pool was divided into aliquots to acquire representative QC samples. QC samples were prepared simultaneously along with study samples and were analyzed throughout the LC-MS analysis sequences every five study samples. Since these samples do not contain any biological variability, they can be considered as technical replicates. For both plasma and urine, study and QC samples were prepared in random order. Blank extracts were prepared simultaneously along with study samples and were analyzed before the LC-MS analysis sequences to check the overall contamination in the analytical pipeline.

Sample preparation for RP-LC Q-TOF-MS based metabolomics

The frozen urine samples were thawed on ice and centrifuged at 20,627 x g for 10 min. Samples were prepared by adding 875 µL of water to 125 µL of urine in Eppendorf tubes and briefly vortex mixing, after which 100 µL was transferred to a vial with glass insert for LC-MS analysis.

The frozen plasma samples were thawed on ice. Subsequently, 50 µL of plasma was transferred to Eppendorf tubes and 200 µL of ice-cold methanol (−20 °C) was added. After vortex mixing during 30 s, samples were placed at -20 °C for 20 min. Afterwards, the samples were centrifuged for 10 min at 20,627 x g and 200 µL of the supernatant was dried in a centrifugal vacuum concentrator. The dried extracts were dissolved in 100 µL of water/acetonitrile 95/5 (v/v) and transferred to a new Eppendorf tube. The samples were centrifuged for 10 min at

20,627 x g, after which 80 µL of supernatant was transferred to a vial with glass insert for LC-MS analysis.

RP-LC Q-TOF-MS based metabolomics

The LC-MS method was adapted from Boelaert *et al.* [1]. The samples (2-µL injection volume) were chromatographically separated on an Acquity UPLC HSS T3 column (2.1 x 100 mm; 1.8 µm; Waters, Milford, MA, USA). Chromatographic separation was achieved on an Agilent 1290 Infinity LC system (Infinity Binary Pump G4220A, Thermostat G1330B, Infinity Sampler G4226A, Infinity Thermostatted Column Compartment G1316C; Agilent Technologies). The column temperature was maintained at 40°C. Eluting buffers were buffer A (0.1% HCOOH in H2O) and buffer B (0.1% HCOOH in acetonitrile). Starting conditions with 0 % buffer B at a flow rate of 0.35 mL/min was held for 1 min. Subsequently, over 15 min a gradient was applied to 100% B followed by an isocratic hold for 4 min before returning to the starting conditions (0% B) for 5 min.

High-resolution accurate mass spectra and fragmentation spectra were obtained with an Agilent 6550 Q-TOF mass spectrometer (MS) (Agilent Technologies) equipped with a Dual Jetstream electrospray ionization (ESI) source. The instrument was operated in both positive and negative electrospray ionization mode. Needle voltage was optimized to +/- 3.5 kV, the drying and sheath gas temperatures were set to 290°C and 400°C and the drying and sheath gas flow rates were set to 13 and 12 L/min, respectively. Data were collected in centroid mode from *m/z* 100–1700 in positive ion mode and *m/z* 100-1700 in negative ion mode at an acquisition rate of 2 spectra/s in the extended dynamic range mode (2 GHz), offering an in-spectrum dynamic range of 105 and a resolution of ± 10000 FWHM in the lipid *m/z* range. To maintain mass accuracy during the analysis sequence, a reference mass solution was used containing reference ions (*m/z* 121.050873 and 922.009798 for positive ESI mode, *m/z* 112.985587 and 1033.988109 for

negative ESI mode). MS/MS experiments were performed in the targeted MS/MS mode, thereby adding precursors of interest in an inclusion list. The quadrupole was operated at narrow resolution (1.3 amu window) and the collision energy was fixed at either 20 or 35 eV. Data acquisition was performed using MassHunter Acquisition B.06.01.

Data analysis was performed using the MassHunter Workstation Software, Profinder, and the Mass Profiler Professional (MPP) Software: MassHunter Qualititative Analysis (Version B.07.00 Build 7.0.7024.29, Service Pack 1), Profinder (Version B.06.00), and MPP (Version 12.1 Build 170166). Raw LC-MS data files were processed in an untargeted fashion using the Molecular Feature Extraction (MFE) algorithm incorporated in the MassHunter Profinder software package. This feature extraction algorithm localizes the unique peaks in the LC-MS chromatogram. Each feature is composed of a retention time, mass and intensity. For comparative metabolomics, the resulting feature files from MFE were imported in MassProfiler Professional 12.0 (Agilent Technologies) which aligned, visualized and filtered the features. For statistical analysis (Mann-Whitney-U test), no filters or normalization steps were performed. Zero values were excluded from the calculation of p-values and fold changes. No multiple testing correction was executed for the statistical analysis, implying the presence of many false positives in the resulting feature lists.

Statistically significant features (p-value < 0.05) containing the appropriate fold change differences were exported and once again extracted from the raw data, a process named recursion. Here, all the extracted ion chromatograms were manually checked, and false positives were excluded. All compounds checked in the recursion step were again subjected to the Mann-Whitney-U test. In these analyses correction for multiple testing was performed with the Benjamini Hochberg false discovery rate.

Quality of analysis of the metabolomics approach

The validity of the performed analyses was monitored in both a targeted and a non-targeted manner using the QC samples. Targeted monitoring was performed by determining the error of the measurement on signal intensity (peak area), retention time and mass accuracy for a list of 18-22 randomly selected metabolites. Peak area fluctuations, originating from both the sample preparation step and the LC-MS analysis, are typically below 15% relative standard deviation [2]. Chromatographic retention time reproducibility is in general satisfactory and less than 1 RSD%. Also, high mass accuracy (< 5ppm) was obtained for all analyses.

Apart from this targeted approach, the reproducibility of the applied metabolomics analysis was examined in a more comprehensive way by calculating the error on all detected features in the QC samples and representing the acquired RSD distribution as depicted in S3 Fig. For all analyses performed, > 75% of all features had an RSD below 30%, which can be defined as the upper limit for untargeted or discovery metabolomics analysis [3].

Metabolite identification

Metabolite identification results from an identification strategy that fully exploits the features of the Q-TOF MS system. Generation of molecular formulas, based on accurate mass, isotope abundance, and isotope spacing both in positive and negative ionization mode, was complemented with accurate mass database searching the Metlin spectral library (Agilent Technologies) and MS/MS measurements in both ionization modes. MS/MS spectra were matched on the Metlin spectral library (Agilent Technologies), compared with literature or interpreted using MassHunter Molecular Structure Correlator (MSC; Agilent Technologies). MSC tries to explain each observed fragment ion into the proposed structure using a "systematic bond-breaking" approach as described by Hill and Mortishire-Smith [4]. Molecular structures from Metlin, Human Metabolome Database (HMDB) and ChemSpider were

imported into MSC, which scores the observed fragment ions from 0-100 to the theoretical fragmentation of the imported structure.

Infrared ion spectroscopy (IRIS) analysis

Details of the hardware modifications to the quadrupole ion trap mass spectrometer and the synchronization with the IR laser are described elsewhere [5]. Ions were generated by electrospray ionization using an Apollo ESI source in positive ion mode. Solutions of the reference standards (1 µM in 50:50 acetonitrile: water) and the unmodified HPLC fraction were introduced at 80-120 µL/h flow rates. The capillary voltage was optimized between -4.4 and -4.6 kV and the nebulizer gas pressure, drying gas flow rate and drying gas temperature were set to 12.3 psi, 4 L/minute and 80 °C, respectively. The generated *m/z* 260 ions were mass-isolated and trapped for 180 ms. The FELIX IR free electron laser was set to produce IR radiation in the form of ~10 µs macropulses at a 10 Hz repetition rate (bandwidth ~0.4% of the centre frequency). The pulse energy varied between 20 and 110 mJ depending on the IR frequency. Stored ions were irradiated by two macropulses. IR photodissociation spectra were recorded over the 800-1900 cm^{-1} region using steps of 3 cm^{-1}. At each IR frequency point, an MS spectrum was recorded after irradiation; each MS spectrum was an average of 6 individual mass spectra. *m/z* 201 daughter ions were generated by collision induced dissociation using a fragmentation amplitude of 0.5 V. These ions were mass-isolated and their IR spectra were also recorded using a procedure similar to that for the precursor ion, but with the IR laser power attenuated by 5 dB to avoid saturation at strong absorptions.

Data analysis was performed using a custom-made script in Bruker Compass DataAnalysis (Version 5.1). This script extracts the intensities of each precursor and fragment ion (center *m/z* +/- 0.3) from the saved MS/MS spectra and calculates the fragmentation yield at each frequency point by relating precursor and fragment intensities (yield = ΣI(fragment ions)/ΣI(precursor +

fragment ions)). The yield was plotted against the IR frequency to generate an IR spectrum. The IR frequency was calibrated using a grating spectrometer, and the IR yield was linearly corrected for frequency-dependent variations in the laser pulse energy.

Synthesis of 2-methyl pentanoyl carnitine and 3-methyl pentanoyl carnitine

Starting material L-carnitine, 2-methyl pentanoyl chloride, 3-methyl pentanoyl chloride, TFA, and Amberlyst® A21 free base resin were purchased from Sigma-Aldrich (Saint Louis, MO, USA). Other reagents and solvents were obtained from VWR International.

^{1}H NMR spectra was acquired on a Bruker 300-Avance (300 MHz) spectrometer with TMS as an internal standard. Chemical shifts were expressed in parts per million (ppm, δ scale). LCMS analysis was performed on an Agilent 6140 Quadrupole LC/MS mass spectrometer and 1200 series HPLC with an Agilent Zorbax® SB C18 column (3.5 □m, 2.1 × 30 mm), and with gradient elution from 10-100% CH_3CN-H_2O containing either 0.1% TFA over 4 min, then held at 100% CH_3CN for 2 min. The flow rate was 0.5 mL/min, UV detection at 214 and 254 nm, mass scan range was 120-1500 amu.

a) 2-Methyl-pentanoyl-L-carnitine synthesis

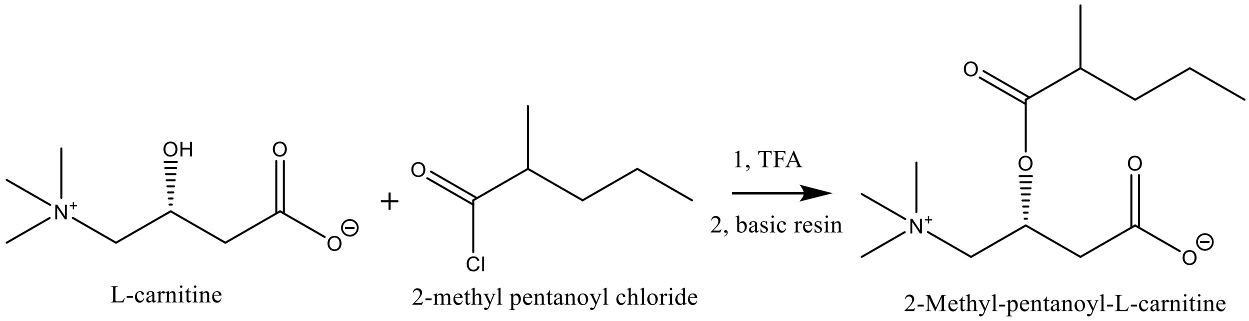

L-Carnitine was dissolved in TFA (4 mL). The solution was added into 2-methyl pentanoyl chloride. The reaction mixture was heated at 50 °C for 12 hrs, and then was evaporated to dryness in vacuo to give a crude product containing TFA as a thick oil.

Part of the of the crude product (thick gum, 888 mg containing TFA) was saved. Remaining crude product (~400 mg) was dissolved in water (5 ml) to give an acidic solution and then was treated with basic resin (Amberlyst® A21 free base resin) until pH became neutral (pH 6-7). The solution was then filtrated and evaporated to give a thick colorless gum, and then foam (334 mg).

HPLC chromatographic Purity was 95% by area normalization of the detected peaks at a wavelength of 215 nm on an Agilent 1100 series HPLC analysis with a Waters XBridge C18 5um 250X4.6mm column; Mobile phase: A: 0.1% TFA in water, B: 0.1% TFA in CH_3CN; isocratic at 10%B in 0-2 min, gradient from 10%B to 20%B in 2-15 min, isocratic at 90%B in 15-17 min, gradient from 90%B to 10%B in 17 to 18 min, isocratic at 10%B in 18-20 min, post run 3 min; flow rate 1 mL/min, injection volume 5 uL of methanol solution at 4 mg/ml; Column temperature 30°C. Retention time Tr = 9.7 min.

LC-MS analysis, m/z 260 ($M+H^+$).

^{1}H NMR ($CDCl_3$) δ 5,60 (m, 1H), 4.05 (m, 1H), 3.75 (m, 1H), 3.30 (s, 9H), 2.60 (m, 1H), 2.45 (m, 2H), 1.60 (m, 1H), 1.30 (m, 3H), 1.10 (t, 3H), 0.90 (t, 3H).

b) 3-Methyl-pentanoyl-L-carnitine synthesis

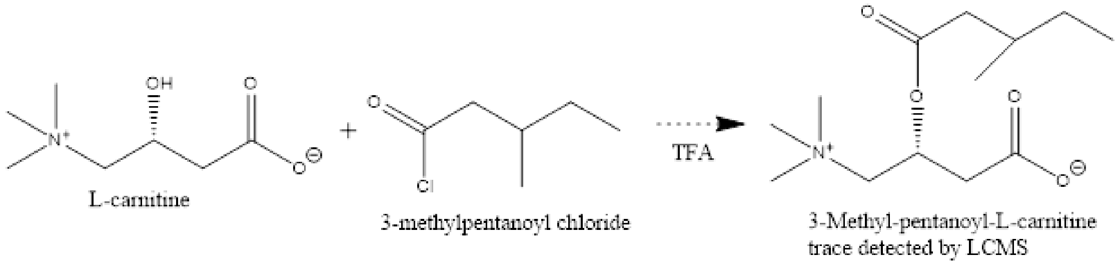

Synthesis of 3-MPC was performed similar to 2-MPC. Resulting reaction mixture was shown to contain traces of 3-MPC based on LCMS analysis.

Supplementary References

1 Boelaert, J. *et al.* State-of-the-art non-targeted metabolomics in the study of chronic kidney disease. *Metabolomics* **10**, 425-442, doi:10.1007/s11306-013-0592-z (2014).

2 Dunn, W. B., Wilson, I. D., Nicholls, A. W. & Broadhurst, D. The importance of experimental design and QC samples in large-scale and MS-driven untargeted metabolomic studies of humans. *Bioanalysis* **4**, 2249-2264, doi:10.4155/bio.12.204 (2012).

3 Spagou, K. *et al.* HILIC-UPLC-MS for exploratory urinary metabolic profiling in toxicological studies. *Analytical chemistry* **83**, 382-390, doi:10.1021/ac102523q (2011).

4 Hill, A. W. & Mortishire-Smith, R. J. Automated assignment of high-resolution collisionally activated dissociation mass spectra using a systematic bond disconnection approach. *Rapid Communications in Mass Spectrometry* **19**, 3111-3118, doi:10.1002/rcm.2177 (2005).

5 Martens, J., Berden, G., Gebhardt, C. R. & Oomens, J. Infrared ion spectroscopy in a modified quadrupole ion trap mass spectrometer at the FELIX free electron laser laboratory. *The Review of scientific instruments* **87**, 103108, doi:10.1063/1.4964703 (2016).
